# Mitochondrial myopathy presenting as fibromyalgia: a case report

**DOI:** 10.1186/1752-1947-6-55

**Published:** 2012-02-10

**Authors:** Mishal Abdullah, Sahana Vishwanath, Amro Elbalkhi, Julian L Ambrus

**Affiliations:** 1 Department of Medicine, SUNY at Buffalo School of Medicine, 100 High Street, Buffalo, NY 14203, USA

## Abstract

**Introduction:**

To the best of our knowledge, we describe for the first time the case of a woman who met the diagnostic criteria for fibromyalgia, did not respond to therapy for that disorder, and was subsequently diagnosed by biochemical and genetic studies with a mitochondrial myopathy. Treatment of the mitochondrial myopathy resulted in resolution of symptoms. This case demonstrates that mitochondrial myopathy may present in an adult with a symptom complex consistent with fibromyalgia.

**Case presentation:**

Our patient was a 41-year-old Caucasian woman with symptoms of fatigue, exercise intolerance, headache, and multiple trigger points. Treatment for fibromyalgia with a wide spectrum of medications including non-steroidal anti-inflammatory drugs, antidepressants, gabapentin and pregabalin had no impact on her symptoms. A six-minute walk study demonstrated an elevated lactic acid level (5 mmol/L; normal < 2 mmol/L). Biochemical and genetic studies from a muscle biopsy revealed a mitochondrial myopathy. Our patient was started on a compound of coenzyme Q10 (ubiquinone) 200 mg, creatine 1000 mg, carnitine 200 mg and folic acid 1 mg to be taken four times a day. She gradually showed significant improvement in her symptoms over a course of several months.

**Conclusions:**

This case demonstrates that adults diagnosed with fibromyalgia may have their symptom complex related to an adult onset mitochondrial myopathy. This is an important finding since treatment of mitochondrial myopathy resulted in resolution of symptoms.

## Introduction

Fibromyalgia is a condition characterized by chronic widespread pain and fatigue which affects approximately 2% of the US population [1]. The diagnostic criteria include pain lasting for longer than three months on both sides of the body, involving the upper and lower halves of the body, and presence of at least 11 out of 18 specific tender points over the body [2].

Over the years, several pathogenic mechanisms for the condition have been postulated but the exact pathogenesis still remains a mystery. Historically, disordered sleep [3], circadian rhythm abnormalities [4], and hormonal imbalance [5] have been looked upon as potential causes, but whether these factors are important in the pathogenesis, or merely arise secondary to bodily stress remains unclear. Alteration in pain modulatory neuropeptides [6] is another widely studied hypothesis, but several studies have shown variable results with no definitive conclusion. Finally, another more recent theory is the presence of underlying muscle metabolic disease that leads to abnormalities in high-energy phosphate metabolites. Other postulated causative explanations include genetic abnormalities, psychiatric disorders, and environmental stressors [7].

Furthermore, due to our lack of understanding of this condition, treating fibromyalgia has always been a challenge for most clinicians. Subsequently, several different groups of medications have been tried over the years. Historically, tricyclic antidepressants have been the most commonly used medications and although short-term studies did show some benefit in some patients, long-term prospective studies failed to reveal any significant effect [8].

Other medication groups used to treat fibromyalgia include selective serotonin reuptake inhibitors (SSRIs) and serotonin-norepinephrine reuptake inhibitors (SNRIs), anticonvulsants, opioid analgesics, sedatives, hypnotics and anti-inflammatories, and although variable responses have been observed, the benefits have been largely unsatisfactory, and patients are all too often exposed to the numerous, potentially lethal side effects of common medications. Generally, only a small group of patients respond, and most people do not have long-term benefit [9].

We present the case of a patient in whom the symptoms of fibromyalgia were related to an underlying mitochondrial disorder. Treatment of the mitochondrial disorder resulted in resolution of symptoms.

## Case presentation

Our patient was a 41-year-old Caucasian woman who presented to our Rheumatology clinic for evaluation of progressive exercise intolerance, fatigue, diffuse myalgias, arthralgias and difficulty sleeping. The pain primarily involved her entire back and arms, and she reported multiple tender points all over her body. On the basis of her symptoms, our patient had also been diagnosed with fibromyalgia, and was treated with multiple different medications without any relief.

Her other medical history included hypothyroidism, cervical disc disease, hypertension and Raynaud's disease. Notable family history included breast carcinoma and hypertension in her mother, and lymphoma in her father. Her medications at presentation included lisinopril 5 mg daily and levothyroxine 25 μg daily. Other pertinent medications she had previously used included pregabalin, amitriptyline and gabapentin. Her physical examination results were normal except for mild tenderness to palpation along her upper and lower back, and shoulders.

Significant laboratory results included creatine kinase (CK) = 325 U/L (normal range 0 to 150 U/L), normal comprehensive metabolic panel (CMP) and complete blood count (CBC), liver function tests and thyroid functions tests. A six-minute walk test revealed a normal resting lactic acid = 1.6 mmol/L, and an elevated post-six-minute-walk lactic acid = 5 mmol/L (normal result < 2 mmol/L). Her ammonia levels were normal at rest and after six-minute walk (14 μmol/L and 38 μmol/L respectively; normal 0 to 40 μmol/L). A muscle biopsy was performed for biochemical evaluation, which revealed several abnormalities including decreased levels of citric acid synthase (49% of normal), cytochrome *c *oxidase (53% of normal), succinate dehydrogenase (72% of normal), and nicotinamide adenine dinucleotide (NADH) dehydrogenase (73% of normal), thereby demonstrating a defect in the mitochondrial respiratory chain. Genome sequencing was performed, which revealed multiple POLG1 polymorphisms (C-T polymorphism at 2254, and G-T polymorphism at 3708) and several mitochondrial genome polymorphisms (1438 A-G, 3992 C-T, 14365 C-T, 14582 A-G, and 4042 A-G).

Our patient was started on a compound of Co-Q10 200 mg, creatine 1000 mg, carnitine 200 mg and folic acid 1 mg to be taken four times a day. She gradually showed significant improvement in her symptoms over a course of several months.

## Discussion

Mitochondrial myopathies are disorders characterized by morphological abnormalities of muscle mitochondria. Accumulating evidence suggests that mitochondrial disorders are among the most common inherited metabolic diseases [10]. Similar to fibromyalgia, patients may present with muscle weakness, pain, fatigue and exercise intolerance that progressively worsens over time.

Several steps are involved in Adenosine-5'-triphosphate (ATP) generation in the mitochondria, and defects in any part of the cycle may impair energy production leading to symptoms [11]. These abnormalities in generation and utilization of ATP can be assessed by specific tests, which as in our patient pointed towards problems in energy metabolism [12]. Genetic testing with sequencing of the mitochondrial genome and chromosomal genes affecting mitochondrial function may also be pursued, as was performed in our patient. Mutations in POLG1 and several mitochondrial genome polymorphisms were noted.

Subsequently, our patient was started on a regimen of coenzyme Q10 (Co-Q10; ubiquinone), creatine, carnitine, folic acid and α-lipoic acid. Co-Q10 transports electrons between complex I and complex III of the mitochondrial respiratory chain and has been shown to improve mitochondrial function in several studies [13]. Creatine generates additional ATP through the creatine phosphate shuttle. Carnitine enhances transport of fatty acids into the mitochondria. Folic acid is a cofactor for several mitochondrial enzymes, while α-lipoic acid is a strong antioxidant [14]. Although this treatment regimen was started several years after symptom onset, within the first few months our patient showed tremendous improvement. With continued therapy, her complaints dissipated over several months, with a gradual but sustained resolution of all symptoms.

## Conclusions

This case postulates the possible role of mitochondrial disease in the pathogenesis of the symptom complex known as fibromyalgia, whereby not only is the underlying defect identified at the molecular and genomic level, but with appropriate therapy, significant symptomatic improvement is also noted.

Underlying mitochondrial disease may not be the only explanation for such a symptom complex, but the exact role of mitochondrial myopathy in the development of fibromyalgia needs to be studied further for a better understanding of the disease, and to ensure adequate and effective patient care. All patients with fibromyalgia should be evaluated for sleep disorders, endocrine disorders such as hypothyroidism and metabolic disorders before a diagnosis of primary fibromyalgia is given. The relative frequency of these medical problems in patients currently diagnosed with fibromyalgia is unclear, but would be worthy of future study.

### Patient's perspective

I am pleased to contribute to this article as to help others with a similar problem. I am truly grateful to Dr Julian Ambrus Jr and to those who referred me to his expert care.

I am a 44-year-old woman with mitochondrial myopathy confirmed by blood tests and muscle biopsy. This disease grew worse as I aged and was exacerbated by pregnancy and other stressors. Certain medications really made me feel ill (which I now know to be mitochondrial killers). My main symptoms included muscle and joint pain, weakness, fatigue, muscle twitching, pain, headaches, and visual disturbances.

I have been to several doctors and have had several tests to rule out certain diseases. This process was slow and frustrating knowing I was sick and not having a clear answer. I was diagnosed with fibromyalgia, but there were other symptoms as well. My feet, hands and legs were weak to where I fell several times down stairs and in my home. I was too weak to hold dishes and to wash them. Simple daily chores proved difficult, and at times I could not get out of bed or a chair without help. The fibromyalgia medicines did not help. As my symptoms waxed and waned, time and rest helped a bit. I was concerned that in time I would get progressively worse.

Today however, I am happy to say that I am feeling much better and able to enjoy normal activities again and more. I take the prescribed treatments for mitochondrial myopathy and can tell you they do work and they have been every bit worth the price.

### Consent

Written informed consent was obtained from the patient for publication of this case report and any accompanying images. A copy of the written consent is available for review by the Editor-in-Chief of this journal.

## Competing interests

The authors declare that they have no competing interests.

## Authors' contributions

JLAJr made the diagnosis and managed our patient. MA, SV and AE participated in the care of our patient at various clinic visits. All authors participated in the writing of this case report. All authors read and approved the final manuscript.
